# Clinical and genomic characterization of hypervirulent *Klebsiella pneumoniae* (hvKp) infections *via* passive surveillance in Southern California, 2020–2022

**DOI:** 10.3389/fmicb.2022.1001169

**Published:** 2022-10-14

**Authors:** Edwin Kamau, Elizabeth L. Ranson, Allison T. Tsan, Elke S. Bergmann-Leitner, Omai B. Garner, Shangxin Yang

**Affiliations:** ^1^Department of Pathology and Laboratory Medicine, UCLA David Geffen School of Medicine, Los Angeles, CA, United States; ^2^Division of Infectious Diseases, UCLA David Geffen School of Medicine, Los Angeles, CA, United States; ^3^Biologics Research and Development, Walter Reed Army Institute of Research, Silver Spring, MD, United States

**Keywords:** hypervirulent *Klebsiella pneumoniae* (hvKP), genomic epidemiology, surveillance, Southern California (United States), invasive infections

## Abstract

Hypervirulent *Klebsiella pneumoniae* (hvKp) is more invasive and virulent than classical *K. pneumoniae*, and requires specialized treatment. To raise clinical awareness, this study determined the prevalence, clinical characteristics, and genomic epidemiology of hvKp infections in Southern California (SoCal) by conducting a passive surveillance in a single large academic medical center. We report here that hvKp infections were more common than expected, accounting for 2.6% of invasive *K. pneumoniae* infections, and presented with a wide disease spectrum, occasionally mimicking tumors, even co-infecting a COVID-19 patient. Most infections were community acquired with no recent international travel, suggesting hvKp strains are circulating in the community. Genomic analysis revealed genetic diversity, with the K1-ST23 lineage predominating but not clonal, and multiple sequence types of K2 including a SoCal unique K2-ST66 sublineage that had been unrecognized. Our findings highlight the urgency of heightened awareness of hvKp infection in the US, the need for rapid diagnosis of hvKp, and the necessity of implementing robust surveillance programs for hvKp at the institutional or local level.

## Introduction

Hypervirulent *Klebsiella pneumoniae* (hvKp) strains cause invasive infections typically characterized by hepatic abscess in the absence of biliary tract disease with metastatic spread. These infections are usually associated with severe complications and may require source control and targeted and/or prolonged treatment (Russo and Marr, 2019; Choby et al., 2020). The symptoms of hvKp are non-specific and may include fevers, chills, abdominal pain, nausea, and vomiting, but may also be specific to the location of the infection (Choby et al., 2020). The disease presentations of hvKp are diverse and distinct from infections caused by the classical *K. pneumoniae* (cKp) (Pomakova et al., 2012). In addition to the typical hepatic infections, common sites of primary and metastatic infections include meningitis and epidural abscesses, endophthalmitis, pneumonia, bacteremia, and necrotizing skin, soft tissue, and bone infections (Kashani and Eliott, 2013; Russo and Marr, 2019; Choby et al., 2020). Less common extrahepatic infections include renal abscess, mycotic aneurysm, septic arthritis, and uterine abscess (Ku et al., 2008; Kamau et al., 2022).

Unlike cKp, which primarily causes opportunistic nosocomial infections in hosts with comorbidities, immunocompromise, or existing barrier breakdown, infections caused by hvKp are most commonly community-acquired and are found in otherwise healthy hosts (Lin et al., 2010; Russo and Marr, 2019; Choby et al., 2020). However, a recent hospital-based study in the US found that even though hvKp infections were mostly community-acquired, many patients had numerous comorbidities and were immunocompromised (Parrott et al., 2021). This contrasts with what has been described in Asia where patients tend to be younger and immunocompetent (Lee et al., 2006; Siu et al., 2012).

Most hvKp strains express highly mucoid capsules, leading to hypermucoviscous colony phenotype, which was historically used to identify hvKp by a positive String test (when a viscous string >5 mm in the length of a bacterial colony is stretched by a bacteriological loop) (Fang et al., 2004). However, the String test can be problematic, since some cKp strains can be hypermucoviscous, and the definition of hvKp may vary (Russo et al., 2015, 2018; Catalán-Nájera et al., 2017; Yang et al., 2020). For a definitive diagnosis, genetic determinants of hypervirulence present on large virulence plasmids and/or chromosomal mobile genetic elements have been used to accurately identify hvKp strains by whole genome sequencing (WGS) or by PCR (Lin et al., 2012; Bialek-Davenet et al., 2014; Struve et al., 2015; Russo et al., 2018; Kamau et al., 2021). These genetic markers include aerobactin siderophore biosynthesis (*iuc*), salmochelin biosynthesis (*iro*), metabolic transporter (*peg-344*), and regulator of mucoid phenotype A (*rmpA*) (Russo et al., 2014, 2015, 2018; Bulger et al., 2017; Harada and Doi, 2018; Choby et al., 2020). Further, comparative genomic studies have shown that hvKp infections are caused by a small number of clonal group lineages, with the K1 capsule-producing serotype being the most dominant lineage (Bialek-Davenet et al., 2014; Struve et al., 2015). This lineage includes sequence type (ST) 23, the leading cause of liver abscesses and the subsequent metastatic infections (Russo and Marr, 2019; Choby et al., 2020). Another important hvKp lineage is the K2 capsule-producing serotype, with ST86 and ST380 predominating (Bialek-Davenet et al., 2014).

Although hvKp is mainly endemic in Asian countries, the incidence of hvKp infection is increasing in the US and other non-endemic regions. Since first reported in the US more than 20 years ago (Saccente, 1999), several reports have been published (McCabe et al., 2010; Kashani and Eliott, 2013; Prokesch et al., 2016; Kamau et al., 2021, 2022), including a surveillance study in a New York City hospital showing 3.7% (17/462) of invasive *K. pneumoniae* (Kp) infections were caused by hvKp stains (Parrott et al., 2021). HvKp infections are likely underdiagnosed and/or misdiagnosed in the US, as demonstrated by recent case studies (Kamau et al., 2021, 2022), and the extent and the epidemiology of hvKp infections in the vast majority of the US patient population are unknown. As an emerging infection, it is important for clinicians and laboratorians to be aware of the possibility of hvKp in their patient populations, and to be able to identify these strains promptly to ensure appropriate treatment (Chung et al., 2007; Tsai et al., 2008; Rossi et al., 2018; Russo and Marr, 2019; Choby et al., 2020; Kamau et al., 2021, 2022). To better understand the prevalence, clinical characteristics, and genomic epidemiology of hvKp infections in Southern California (SoCal), we conducted passive surveillance of hvKp infections in our patient population based on both clinical suspicion and laboratory definitive identification using WGS.

## Materials and methods

### Patients and specimens

The hvKp cases were passively identified in our institution from September 2020 to March 2022. Specimens including blood, tissue, body fluid, sputum, endotracheal aspirate, bronchoalveolar lavage (BAL), and abscess/drainage were collected from patients admitted to the UCLA Health System and submitted to the UCLA Clinical Microbiology Laboratory for identification as part of standard care. The passive surveillance was mainly based on laboratory findings of hypermucoviscous Kp isolates from non-urine and non-superficial wound sources, and partially (*n* = 5) coupled with clinical suspicion from providers. Inadvertently, all five cases with clinical suspicion were hypermucoviscous. The clinical suspicion for hvKp included at least one of the following findings: (1) hepatic or extrahepatic abscess, (2) head and neck infections, and (3) patients with diabetes. The laboratory suspicion was mainly based on the hypermucoviscous phenotype and positive String test. The overall invasive Kp infection case number was counted by cases in which Kp was isolated from any of the sterile clinical samples, including blood (32.6%), tissue (4.8%), body fluid (14.3%, including liver aspirate and abdominal drainage), as well as non-sterile but critical anatomical sites including lower respiratory samples (39.2%, including sputum, endotracheal aspirate, BAL), eye specimens (0.4%), and drainage/abscess from the head and neck areas (8.8%). Cases of Kp isolated from urine and superficial wounds/lesions were excluded. Only one isolate per patient was included in the study. This study was reviewed by the UCLA Human Research Protection Program and received an IRB exemption. Table 1 provides a summary of demographics and clinical presentation of cases. The detailed clinical history is given in Supplementary Table S1.

**Table 1:** Summary of hvKp cases.

**Case**	**A**	**S**	**R**	**Clinical impression**	**Comorbidities**	**Outcomes**
1	76	F	A	Hepatic abscesses, secondary empyema, and bacteremia. Abscess drained.	A-Fib, TBAD, HTN, HT	DRF, then DH
2	64	F	A	Hepatic abscess, urinary tract infection, and bacteremia. Abscess drained.	HBV, CRC s/p chemo (10 years ago)	DH
3	52	F	A	Pyelonephritis and hepatic abscess. Abscess drained.	None	DH
4	76	F	A	Orbital cellulitis with posterior globe abscess. Abscess drained.	cirrhosis, HCC	Evisceration - DH
5	43	M	W	Trauma with respiratory colonization.	Meth	DRF^*^
6	56	M	W	Recurrent pneumonia.	Stage IV LAC	Outpatient
7	48	M	H	Neck abscess. Abscess drained.	T2DM, DKA, SP	DH
8	52	F	H	Pelvic abscess with bacteremia. Abscess drained.	T2DM	DH
9	79	F	B	Pneumonia.	COPD, CVA, HTN	Expired
10	82	M	A	Hepatic and perigastric micro-abscesses	None	DH
11	59	M	W	Presumed hepatic abscess with pylephlebitis and bacteremia. Attempt to drain abscess failed.	CVA, GERD	DH
12	30	M	W	Infection of right eye. Left parotid abscess and right ear drained.	T1DM, DKA, Meth	DRF
13	50	M	B	Cavitary pneumonia in the setting of severe COVID-19.	COVID-19	Expired
14	39	M	A	Hepatic abscess with bacteremia. Abscess drained.	None	DH
15	55	F	H	Necrotizing pneumonia with empyema, erosive abscess, and perinephric abscess. Abscess drained.	T2DM, HTN	DH

A, age; S, sex; R, race, Hispanic (H), White (W), Asian (A), Black (B); T2DM, type 2 diabetes mellitus; T1DM, Type 1 diabetes mellitus; HCC, hepatocellular carcinoma; Meth, methamphetamine use; CRC, colorectal cancer; TBAD, Type B aortic dissection; A-Fib, atrial fibrillation; HTN, hypertension; HT, hypothyroidism; CVA, cerebrovascular accident; DKA, diabetic ketoacidosis; SP, sepsis; GERD, gastroesophageal reflux disease; LAC, lung adenocarcinoma; Race (R); DH, discharge home, no residual deficits; DRF, discharged to a rehabilitation facility. Three patients (20%) endorsed international travel in the last 5 years. All patients were treated with antibiotics.

* Patient #8 required rehabilitation due to his trauma, not the complication of infection.

### Bacterial identification and antimicrobial susceptibility testing

Samples were inoculated on the sheep blood agar plates (Becton Dickinson, MD, USA) and incubated at 35°C in 6–10% CO_2_ for 12 to 24 h. Based on our standard laboratory operating procedures, bacterial isolates were identified to species level using matrix-assisted laser desorption/ionization time-of-flight mass spectrometry (MALDI-TOF, VITEK MS, BioMérieux, NC, USA), which does not distinguish Kp from other species or subspecies in the Kp complex, such as *K. quasipneumoniae*. Drug minimal inhibitory concentrations (MICs) were determined by broth microdilution following CLSI guidelines per UCLA's established protocol.

### String test

The String test was performed as previously described (Fang et al., 2004). In brief, a fresh bacterial colony on a plate was scooped with an inoculation loop and stretched out. Those that formed a viscous string >5 mm in length were considered “String test positive.” The String test was performed at the request of the treating team based on clinical suspicion or based on laboratory findings of apparent hypermucoviscosity of Kp isolates.

### Genetic analysis of the bacterial isolates

The Qiagen EZ1 Blood and Tissue Kit and the EZ1 Advanced XL instrument were used to extract genomic DNA from pure Kp isolates with a positive String test. Extracted DNA was quantified with the Qubit^TM^ 1X dsDNA HS assay using the Qubit^TM^ 3.0 Fluorometer (ThermoFisher Scientific, Waltham, MA). Acceptable quantities of DNA were ≥0.04 ng/μl. DNA was diluted in water to obtain concentrations within the range of 100 to 500 ng in 30 μl. Library preparation was performed using the Nextera DNA Flex Library Prep Kit (Illumina) according to the manufacturer's instructions. The Illumina MiSeq System was used to produce 250 bp paired-end reads. Data were uploaded to the Illumina BaseSpace cloud and de-multiplexed. The sequence files were submitted to the National Center for Biotechnology Sequence Read Archive (https://www.ncbi.nlm.nih.gov/sra) under BioProject accession no. PRJNA729785. The sequences were analyzed using the CLC Genomics Workbench (QIAGEN, https://www.qiagen.com) and Geneious Prime (Biomatters, New Zealand). The paired raw FASTQ sequences of each of the isolates were processed by the CLC *Trim Reads* tool with the default setting and mapped against reference genomes of hvKp strains or plasmids. Consensus sequences were generated by the CLC *de novo Assembly* tool with the default setting. K-*mer* phylogenic analysis was performed using the *Create K-mer Tree* tool on CLC Genomics Workbench using the consensus sequence for each of the isolates. K-mer's of 16 nucleotide lengths were used, and Jensen–Shannon Divergence was used to constructing the phylogenetic trees. Single Nucleotide Polymorphism (SNP) analysis was performed on CLC Genomics Workbench. Variant tracks used in SNP analysis were generated using different reference genomes based on the serotype of the strains; for K1 serotype, NTUH-K2044 (GenBank accession AP006725) was used and for K2 serotype, Kp52.145 (GenBank accession FO834906) was used. The multilocus sequence types (MLST), virulence factors, and K type were determined using BIGSdb-Pasteur (http://bigsdb.pasteur.fr/klebsiella), the genomic-based strain taxonomy, and nomenclature platform of Institut Pasteur. The Center for Genomic Epidemiology (CGE) bioinformatics web service (https://cge.cbs.dtu.dk/services) was used as follows: ResFinder for antimicrobial resistance genes, PlasmidFinder for the presence of plasmid(s) or replicon indicating the presence of plasmid(s), and CSI Phylogeny 1.4 for SNP calling inferring Phylogeny.

A Kp isolate was considered hvKp when at least three or more of the following virulence genes are detected with >99% full-length gene coverage: *iucA, iroB, peg-344, rmpA*, and *rmpA2* (Russo et al., 2018). For this analysis, briefly, the raw sequence reads of each sample were mapped to a concatenated reference genome comprising the five full-length genes using the CLC Genomics Workbench *Map Reads to Reference* tool with the default setting. A gene is considered detected when the average coverage is >15X and the fraction of reference covered is >99%.

## Results

### Patient demographics and clinical characteristics

Between Fall 2020 and Spring 2022, out of 567 invasive Kp infections, we identified 15 (2.6%) hvKp infections. Patients ranged in ages from 30 to 82 years (median age 55), 40% (*n* = 6) were Asian, 27% (*n* = 4) White, 20% (*n* = 3) Hispanic, and 13% (*n* = 2) Black. About 46% (*n* = 7) were female and only 20% (*n* = 3) endorsed international travel in the last 5 years (Table 1). Most (80%, *n* = 12) had comorbidities, with diabetes mellitus being the most common (27%, *n* = 4). Most (87%, *n* = 13) of the infections were community-acquired. There were two cases that may have been related to the healthcare setting but colonization of hvKp before admission could not be ruled out: patient #13 was intubated on hospital day 6 for worsening COVID-19 pneumonia, and grew hvKp from endotracheal culture collected on hospital day 8; patient #9 was admitted from a skilled nursing facility with hvKp pneumonia and multi-organ failure. Both patients died. One patient (#12) with endophthalmitis lost vision in one eye. However, most (73%, *n* = 11) patients had a favorable outcome and were discharged with no residual deficits.

Patients presented with a wide variety of chief complaints including abdominal pain, dyspnea, cough, fever, eye pain, flu-like symptoms, neck swelling and pain, inability to urinate, and syncope (Supplementary Table S1). There were six patients (40%) with liver abscesses, one of whom had concurrent cholecystitis, but others did not have an underlying hepatobiliary disease. Other sites of infections included the lung (*n* = 6), the neck (patient #7), the pelvis (patient #8), and the eye (patients #4 and #12). Seven patients (47%) had concurrent bacteremia. In two patients, co-infections likely contributed to the disease process: COVID-19 pneumonia (patient #13) and neck abscess (patient #7) with *Streptococcus anginosus* also growing from culture (Supplementary Table S1). Drainage was performed in all 10 cases of abscess, with exception of patient #2, in whom drainage was attempted but failed (Table 1). Ceftriaxone was the most commonly used antibiotic (60%, *n* = 9). Some patients received additional outpatient treatment with ciprofloxacin or trimethoprim-sulfamethoxazole for an additional 3–4 weeks. Overall, the average length of antibiotic treatment was 5 weeks (range, 2–8 weeks).

### Microbiological and genomic characteristics

**Figure 1:**
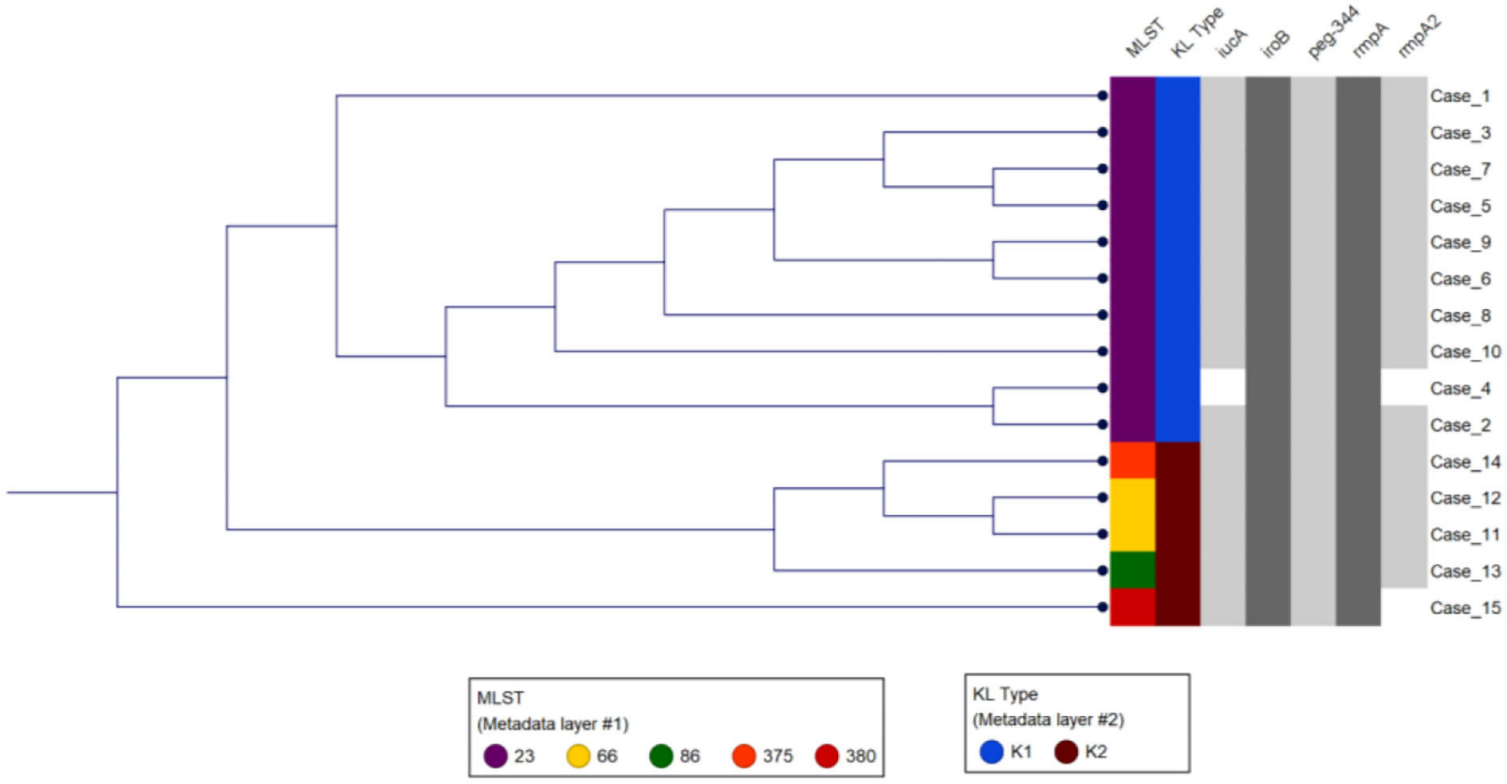
Genetic analysis of the study isolates. The first two columns show the MLST and K type for each isolate. Phylogenetic analysis using K-mer grouped isolates based on the K type and MLST. The gray shaded columns indicate the virulence genes present in each of the isolates.

All isolates were String test positive and susceptible to all drugs tested including piperacillin/tazobactam, cephalosporins, carbapenems, aminoglycosides, fluoroquinolones, and trimethoprim/sulfamethoxazole. Most (67%, *n* = 10) isolates were K1 serotype, and the rest (33%, *n* = 5) K2 serotype (Figure 1). The K1 serotype isolates were closely related to reference strain NTUH-K2044 (GenBank accession AP006725), and the K2 serotype isolates were closely related to reference strain Kp52.145 (GenBank accession FO834906, Supplementary Table S2). All K1 serotype isolates belonged to multilocus sequence type 23 (K1-ST23; Figure 1, Supplementary Table S2), the leading cause of liver abscesses and subsequent metastatic infections (Bialek-Davenet et al., 2014; Choby et al., 2020). Indeed, of the six liver abscesses in our study patients, five were caused by K1-ST23. Of the five K2 isolates, two belonged to the K2 serotype ST66 (K2-ST66; Supplementary Table S2), a sublineage first described a century ago in Indonesia, with only a few other clinical isolates described in the USA, Europe, and Australia (Holt et al., 2015; Rodrigues et al., 2020; Kamau et al., 2021; Klaper et al., 2021; Parrott et al., 2021; Kochan et al., 2022). Comparison of the chromosomal sequence of K2-ST66 isolates in our study to the other four known K2-ST66 strains with available WGS by SNP analysis revealed that UCLA isolates were more genetically related (differed by only 59 SNPs) compared to the strains isolated in Illinois, Europe, and Australia (differed by >750 SNPs), and branched separately, suggesting a unique K2-ST66 sublineage is circulating in SoCal (Figure S1).

**Figure 2:**
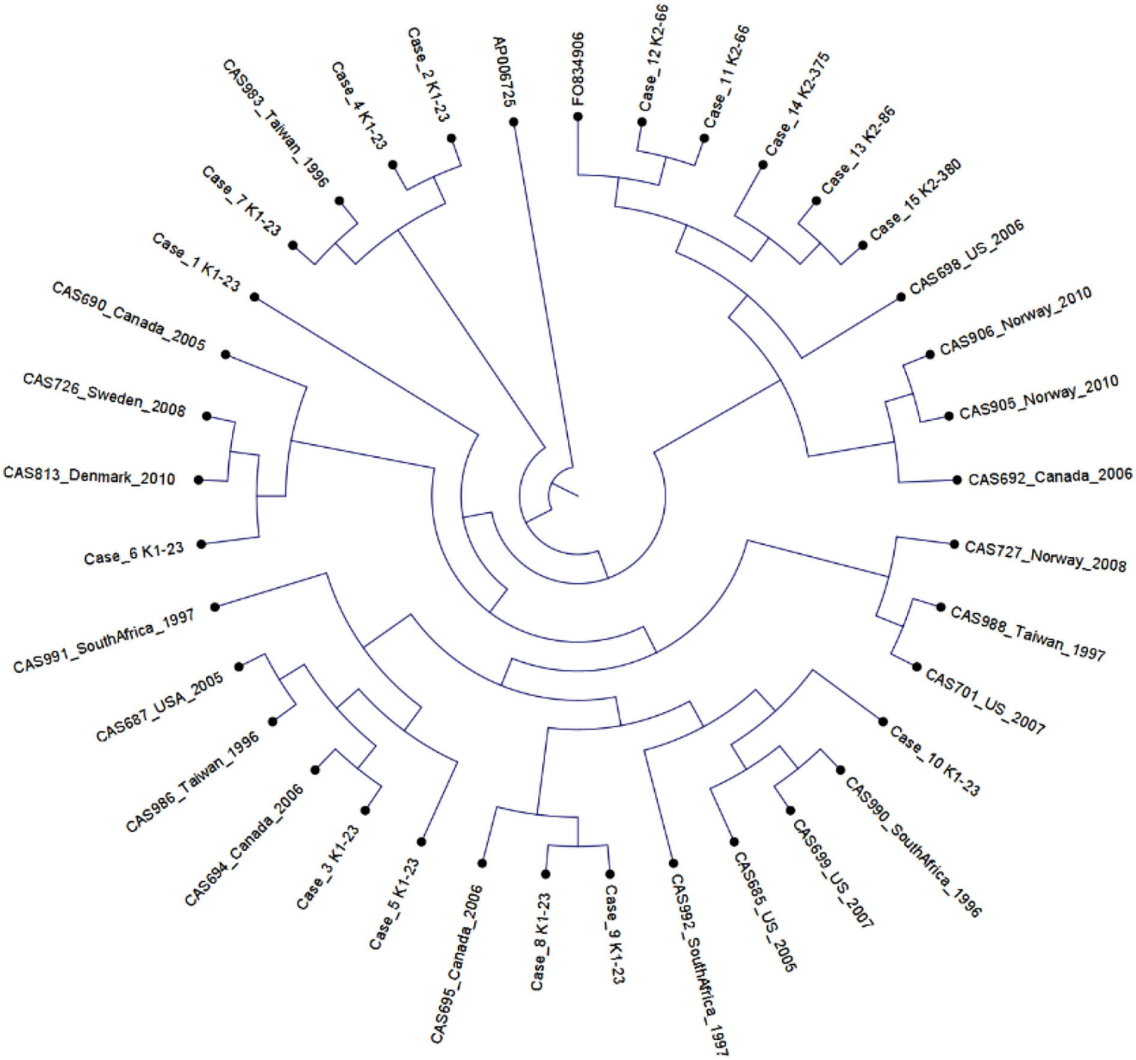
Phylogenetic analysis of 20 K1-ST23 isolates from different geographical origins labeled with country of origin and year of sampling (Lin et al., 2012), 10 K1-ST23 isolates from our study population, and two references strains; K1 serotype (AP006725) and K2 serotype (F0834906).

**Figure 3:**
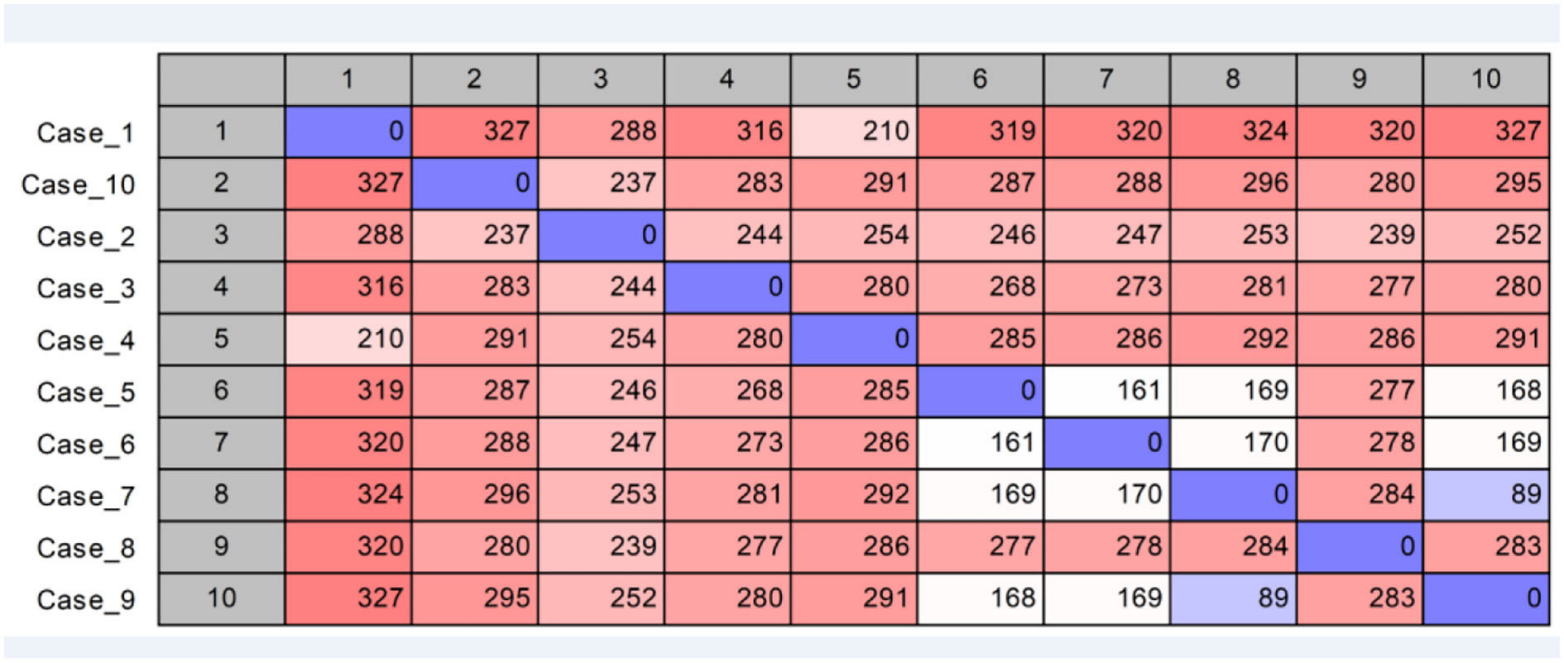
SNP analysis of K1-ST23 isolates using NTUH-K2044 (GenBank accession AP006725) as the reference genome.

Using published hvKp reference genomes from different geographic origins (Struve et al., 2015), we performed K*-mer*-based phylogenetic analysis to better understand the genomic epidemiology of hvKp in our patient population. The phylogenetic tree showed a great genetic diversity of the UCLA hvKp strains compared to the international strains (Figure 2), indicating they were not clonal and had origins from different regions of the world. All isolates clustered based on the K serotype, with the K1 serotype isolates clustering with K1 reference strain (AP006725) and K2 isolates clustering with K2 reference strain (FO834906). We then performed SNP analysis to further assess the genetic relatedness of the 10 K1-ST23 isolates. The mean SNP difference among our isolates was 264 (range 89–327) (Figure 3), similarly to the mean SNP difference among the reference genomes worldwide (240, 55–407) (Supplementary Figure S2), further suggesting that isolates in our study are genetically diverse and not clonal.

### Virulence factors

All 15 hvKp isolates possessed the *peg-344* gene, which is used as a marker for hvKp (Russo et al., 2018). Nine of the 10 K1-ST23 isolates carried a plasmid highly similar to the reference hvKp strain NTUH-K2044 plasmid pK2044 (GenBank accession AP006726) with coverage and pairwise identity >98%, except for UCLA664 (patient #2), which had a lower pairwise identity (93.7%) (Supplementary Table S2). These isolates also carried the plasmid-borne virulence genes including *iucA, iroB, rmpA*, and *rmpA2* genes (Figure 1, Supplementary Table S2). Interestingly, no plasmid was identified in one K1-ST23 isolate, UCLA509 (patient #4), and it only carried *iroB, rmpA*, and *peg-344*, but not *iucA* or *rmpA2*. Indeed, hvKp strains without plasmids have previously been described, most likely due to chromosomal integration of the genetic elements carrying the virulence genes (Ye et al., 2016). It is also likely that UCLA509 has the virulence genes integrated into its chromosome, but additional genomic studies including long-read sequencing are required to confirm this hypothesis. We also cannot rule out plasmid rearrangements that may have occurred that would be missed by PlasmidFinder. All five K2 isolates carried the four virulence genes (*iucA, iroB, rmpA*, and *peg-344*). Of note, the two K2-ST66 isolates [UCLA591 (patient #11) and UCLA353 (patient #12)] carried two plasmids nearly identical to the Kp52.145 plasmids (FO834905 and FO834904) (Supplementary Table S2) and an extra truncated allele of *rmpA* gene on the second plasmid (FO834904 like).

As a comparison, we performed virulence gene analysis in 12 non-hvKp isolates with WGS data available, including 9 with negative String test and 3 with hypermucoviscous phenotype (positive String test). None of the 5 virulence genes (i.e., *iucA, iroB, peg-344, rmpA*, and *rmpA2*) was detected in 11 of them, and only 2 virulence genes (*iucA* and *rmpA*) were detected in one hypermucoviscous isolate from sinus drainage, which was typed as K4-ST90 and not considered hvKp (data not shown).

## Discussion

In recent years, increasing numbers of hvKp infections have been reported in the US (McCabe et al., 2010; Siu et al., 2012; Prokesch et al., 2016; Choby et al., 2020; Kamau et al., 2021, 2022; Parrott et al., 2021; Kochan et al., 2022). This is likely due to multiple factors, including a wider spread of hvKp, increased awareness, and improved diagnosis. In this study, we found the prevalence of hvKp to be 2.6% among our patient population in SoCal, slightly lower than previously reported hvKp prevalence in New York City (3.8%) (Parrott et al., 2021). Notably, in the New York study, the sources of Kp isolates only included blood and body fluid, while in this study, we expand the sources of Kp infections to include the lower respiratory tract and head and neck abscess/drainage. We also observed diverse syndromes in our patient population including abdominal, thoracic, soft tissue, and ophthalmologic diseases. Most infections were community-acquired and our patient population had similar risk factors as those reported in New York City with high comorbidities, immunosuppression, and advanced age (Parrott et al., 2021). Most patients denied any recent international travel, indicating that these hvKp strains are circulating in the community. Although 40% of the hvKp patients were Asian, this is confounded by a large Asian population in SoCal, and thus not strong evidence to suggest that Asians are at increased risk for hvKp infection.

Importantly, we showed that hvKp infection can present with a wide spectrum of extrahepatic presentations including pulmonary abscesses and empyema, soft tissue infection, cavitary pneumonia, orbital cellulitis with posterior globe abscess, endophthalmitis, vitreous abscess, and uterine abscess. Further, we showed that extrahepatic presentations of hvKp infections can be challenging to diagnose and can often mimic malignancy. In two patients (patients #7 and # 8), the initial diagnosis was malignancy (in the neck and uterus, respectively), and it took collaborative efforts of laboratorians and infectious disease clinicians to uncover the true diagnosis. Notably, the extrahepatic infections in our cohort had worse clinical outcomes than the hepatic infections: the two patients who died, both had pneumonia without bacteremia and the patient with endophthalmitis suffered long-term sequela. Notably, one of the deceased patients (#13) had COVID-19 pneumonia and developed new cavitary superinfection with hvKp, which likely contributed to his death. This is the first reported case of hvKp pneumonia in a patient with severe COVID-19 in the US. A case of COVID-19 and fatal sepsis caused by hvKp was reported in Japan in 2020 (Hosoda et al., 2021), and an observational study conducted in an Italian hospital in 2020–2021 reported the spread of multidrug-resistant (MDR) hvKp in patients with COVID-19 that led to a high mortality rate [48.3% (14/29)] (Falcone et al., 2022). As the epidemiology of hvKp expands and infection becomes increasingly endemic in the US, clinicians and laboratorians must recognize invasive disease with or without hepatic manifestation.

Comparative genomic studies have shown that hvKp infections are caused by a small number of lineages, with K1-ST23 predominating. This lineage evolved from a single common ancestor, spreading globally through multiple international transmissions (Bialek-Davenet et al., 2014; Struve et al., 2015). In contrast, the K2 strains are more genetically diverse, with multiple distinct STs, including K2-ST86 and K2-ST380 as the most common lineages (Bialek-Davenet et al., 2014; Struve et al., 2015). Unlike Asia where hvKp infections are predominantly caused by K1-ST23, surveillance studies in North America have shown much larger genetic diversity. In the New York City study, only 24% (4/17) of hvKp infections were K1-ST23, while 45% (8/17) were K2 without dominant sequence type (Parrott et al., 2021). Similarly, in Calgary, Canada, only 29% (4/14) were K1-ST23, the rest were either K2, K5, or non-typeable (Peirano et al., 2013). Our study showed that the hvKp genomic epidemiological pattern in SoCal is more similar to Asia, with K1-ST23 as the predominant lineage accounting for 67% (10/15) of cases, while the K2 lineages were genetically diverse including ST66, ST380, and ST375. Interestingly, we found two cases of K2-ST66 lineage, which is nearly a century old and seldom reported (Holt et al., 2015; Baekby et al., 2018; Rodrigues et al., 2020; Klaper et al., 2021; Parrott et al., 2021; Kochan et al., 2022), suggesting this rare lineage is circulating in our patient population and has gone under-recognized. We also showed that our hvKp isolates are not clonal, which suggests that these isolates did not originate from any one particular geographic region and have been introduced into our patient population from different parts of the world.

The development of antibiotic drug resistance is of great concern to public health. The evolution of MDR and extensively drug-resistant (XDR) hvKp is disconcerting, especially as these strains spread across the world (Russo and Marr, 2019). Studies have demonstrated the expansion of defined clonal groups associated with drug-resistant hvKp, with the evolution toward increasing levels of antimicrobial drug resistance (Bialek-Davenet et al., 2014; Wyres et al., 2020). In our study, all the hvKp isolates were pan-drug susceptible. However, it is to be expected that the expansion of MDR or XDR hvKp to our patient population is inevitable, and we must therefore have a robust surveillance program in place for real-time monitoring and early warning. As of the writing of this manuscript, our laboratory has implemented a real-time WGS test for all Kp isolates with either laboratory suspicion due to a positive String test and/or request from clinicians with clinical suspicions for hvKp infection in their patients. The average turn-around time (TAT) for WGS is 1 week. This is not ideal, but an incremental improvement for hvKp diagnosis. More rapid and cost-effective molecular tests, such as multiplex PCR-based assays are needed to provide faster TAT.

This study has several limitations. First, the suspicion of hvKp was largely based on hypermucoviscosity phenotype, which may lead to missed cases of hvKp without hypermucoviscosity. Therefore, the true hvKp prevalence is likely underestimated. Second, we did not perform long-read sequencing to close the chromosomes and plasmids, which limited our ability to completely characterize the genomics of these hvKp isolates. In addition, this was a passive surveillance study without well-defined clinical criteria for case selection and was conducted in a single health care system. Therefore, our findings may not represent the true epidemiological pattern of hvKp infections in SoCal.

In conclusion, we have described the clinical and genomic characteristics of hvKp infections in Southern California. We report the prevalence and wide disease spectrum (including mimicking malignancy) of hvKp infections. HvKp affects not only immunocompetent people but also very frequently immunocompromised patients with comorbidities. We identified one case of COVID-19 and hvKp co-infections which led to death. HvKp affects not only Asians but also patients of all ethnicity, the majority without significant travel history. It is genetically diverse, with K1-ST23 predominating, but not clonal. We also discovered a unique K2-ST66 sublineage circulating in SoCal that has been unrecognized. Our findings highlight the urgency for heightened awareness of hvKp infection in the US, the need for rapid diagnosis of hvKp, and the necessity of implementing a robust surveillance program for hvKp at the institutional or local level.

## Data availability statement

The datasets presented in this study can be found in online repositories. The names of the repository/repositories and accession number(s) can be found in the article/Supplementary material.

## Ethics statement

The studies involving human participants were reviewed and approved by UCLA Human Research Protection Program. Written informed consent for participation was not required for this study in accordance with the national legislation and the institutional requirements.

## Author contributions

EK, ER, and SY contributed in the design, data collection and analysis, and writing of the manuscript. AT contributed to the data collection and data analysis. EB-L and OG contributed to the revision of the manuscript and providing financial resources for this study. SY also contributed to overall supervision of the study. All authors contributed to the article and approved the submitted version.

## Conflict of interest

The authors declare that the research was conducted in the absence of any commercial or financial relationships that could be construed as a potential conflict of interest.

## Publisher's note

All claims expressed in this article are solely those of the authors and do not necessarily represent those of their affiliated organizations, or those of the publisher, the editors and the reviewers. Any product that may be evaluated in this article, or claim that may be made by its manufacturer, is not guaranteed or endorsed by the publisher.

## Author disclaimer

Material has been reviewed by the Walter Reed Army Institute of Research. There is no objection to its presentation and/or publication. This paper has been approved for public release with unlimited distribution. The investigators have adhered to the policies for protection of human subjects as prescribed in AR 70-25. EB-L and EK are government employees and this work was prepared as part of their official duties. The views expressed in this article are those of the author and do not necessarily reflect the official policy or position of the Department of the Army, Department of Defense, the U.S. Government.
