# Supplementary material for: Clinical and genomic characterization of hypervirulent Klebsiella pneumoniae (hvKp) infections via passive surveillance in Southern California, 2020–2022
Source: Front Microbiol. 2022 Oct 14;13:1001169. doi: 10.3389/fmicb.2022.1001169 (PMC9614223; doi:10.3389/fmicb.2022.1001169)
Supplement: Supplementary file 1 [file Image_1.pdf]

Figure S1. SNP analysis comparing K2-66 isolates, two from our study and four from previously reported strains (30,31,34,35). The variant tracks were generated using the K2 reference strain, Kp52.145 (GenBank accession FO834906). **A.** Phylogenetic tree. **B.** SNP matrix.

A.

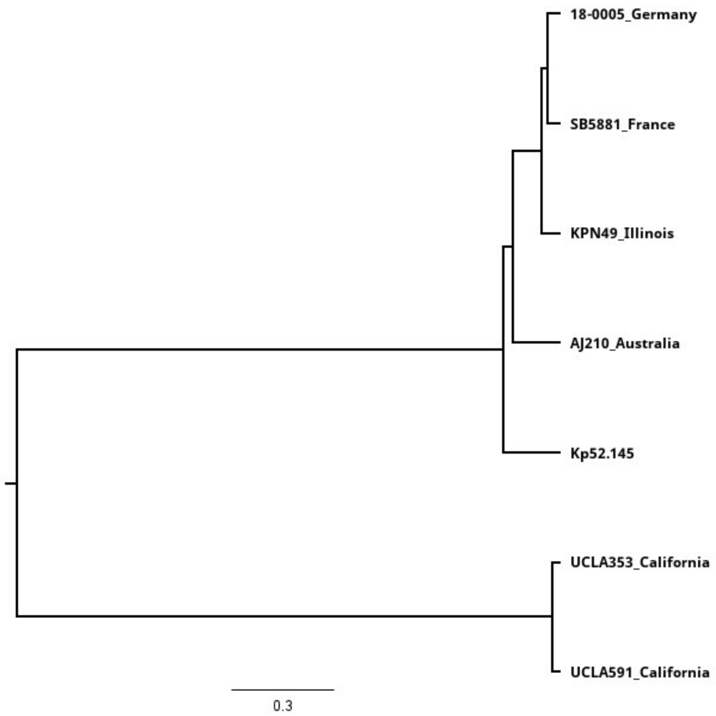

B.

	18-0005_G...	AJ210_Aust...	KPN49_Illin...	Kp52.145	SB5881_Fr...	UCLA353_C...	UCLA591_C...
18-0005_Germany		280	117	279	93	803	798
AJ210_Australia	280		323	451	286	973	968
KPN49_Illinois	117	323		318	136	840	833
Kp52.145	279	451	318		298	756	753
SB5881_France	93	286	136	298		818	813
UCLA353_California	803	973	840	756	818		59
UCLA591_California	798	968	833	753	813	59	
