# Supplementary material for: Clinical and genomic characterization of hypervirulent Klebsiella pneumoniae (hvKp) infections via passive surveillance in Southern California, 2020–2022
Source: Front Microbiol. 2022 Oct 14;13:1001169. doi: 10.3389/fmicb.2022.1001169 (PMC9614223; doi:10.3389/fmicb.2022.1001169)
Supplement: Supplementary file 2 [file Image_2.pdf]

Figure S2. SNP analysis of global reference hvKp isolates using NTUH-K2044 (GenBank accession AP006725) as the reference genome.

		1	2	3	4	5	6	7	8	9	10	11	12	13	14	15	16	17	18	19	20
CAS685	1	0	187	166	321	186	205	168	236	214	203	188	213	287	343	150	188	191	201	163	174
CAS687	2	187	0	194	332	199	216	181	251	229	214	205	226	298	354	177	201	204	209	170	181
CAS690	3	166	194	0	328	193	212	175	243	221	210	195	221	293	350	157	195	198	205	170	181
CAS692	4	321	332	328	0	333	348	315	385	363	346	339	360	318	374	311	335	338	347	308	319
CAS694	5	186	199	193	333	0	217	180	252	230	215	204	227	299	355	176	200	203	214	175	186
CAS695	6	205	216	212	348	217	0	199	269	247	224	221	244	314	370	195	219	222	229	192	203
CAS698	7	168	181	175	315	180	199	0	234	212	197	186	209	281	337	158	182	185	196	155	168
CAS699	8	236	251	243	385	252	269	234	0	150	267	258	237	351	407	228	254	257	265	227	238
CAS701	9	214	229	221	363	230	247	212	150	0	245	236	215	329	385	206	232	235	243	205	216
CAS726	10	203	214	210	346	215	224	197	267	245	0	221	242	312	368	193	217	220	229	190	201
CAS727	11	188	205	195	339	204	221	186	258	236	221	0	233	305	361	178	206	209	220	181	192
CAS813	12	213	226	221	360	227	244	209	237	215	242	233	0	326	382	205	229	232	238	202	213
CAS905	13	287	298	293	318	299	314	281	351	329	312	305	326	0	338	277	301	304	313	272	285
CAS906	14	343	354	350	374	355	370	337	407	385	368	361	382	338	0	331	357	360	369	330	341
CAS983	15	150	177	157	311	176	195	158	228	206	193	178	205	277	331	0	178	181	192	153	164
CAS986	16	188	201	195	335	200	219	182	254	232	217	206	229	301	357	178	0	55	216	173	188
CAS988	17	191	204	198	338	203	222	185	257	235	220	209	232	304	360	181	55	0	219	180	191
CAS990	18	201	209	205	347	214	229	196	265	243	229	220	238	313	369	192	216	219	0	139	150
CAS991	19	163	170	170	308	175	192	155	227	205	190	181	202	272	330	153	173	180	139	0	111
CAS992	20	174	181	181	319	186	203	168	238	216	201	192	213	285	341	164	188	191	150	111	0
