# Supplementary material for: Clinical and genomic characterization of hypervirulent Klebsiella pneumoniae (hvKp) infections via passive surveillance in Southern California, 2020–2022
Source: Front Microbiol. 2022 Oct 14;13:1001169. doi: 10.3389/fmicb.2022.1001169 (PMC9614223; doi:10.3389/fmicb.2022.1001169)
Supplement: Supplementary file 3 [file Table_1.docx]

**Table S1.** Detailed clinical history of the hvKp cases.

| **Case#** | **Impression** | **Presentation** | **Clinical course** |
| --- | --- | --- | --- |
|  |  |  |  |
| 15 | Necrotizing pneumonia with empyema, erosive abscess, and perinephric abscess | A 55-year-old woman with a history of poorly controlled type two diabetes mellitus and hypertension presented with productive cough over 24 hours and acute onset of dyspnea. CT of the chest showed multifocal pneumonia of all lobes with dense consolidations and surrounding nodularity, as well as necrotic cavitation in the left lower lobe, extending through the diaphragm into the left upper quadrant of the abdomen and involving the superior pole of the left kidney, and measuring at least 8.4x4.7x9.7cm. Family reported intermittent altered mental status at home over the last few months prior to presentation. | Patient was intubated and required vasopressors. The abscess was drained (300cc) percutaneously. Pigtail catheter was left in place over the next 3 weeks.  Peripheral blood cultures and sputum collected in the ED grew Kp, *C. albicans* and *C. neoformans*. Patient was treated with ceftriaxone and metronidazole for 14 days followed by 3 weeks of ciprofloxacin for a total 5-week course. She made a full recovery. |
| 11 | Presumed hepatic abscess with pylephlebitis and bacteremia | A 59-year-old man with a history of, gastroesophageal reflux disease and remote splenectomy presented with acute onset of fevers, chills, and nausea over 24 hours. He endorsed nausea, nonbilious emesis, dyspnea, fatigue, and epigastric pain radiating to his back. He was found to be in severe sepsis and had leukocytosis, mild hyponatremia, acute kidney injury and elevated transaminases. On CT the liver was enlarged, measuring approximately 22.0 cm in a craniocaudal dimension with large masses within the left hepatic lobe. The largest mass measured 10.8 cm in greatest dimension with an adjacent 6.9 cm mass and smaller masses seen throughout the remainder of the right hepatic lobe. Thrombosis of the left hepatic vein was also present. | Patient was admitted to the ICU and started on meropenem and vancomycin, which were switched to ceftriaxone 2g daily after blood cultures grew hvKp. On hospital day 7 he underwent esophagogastroduodenoscopy (EGD) with ultrasound guided fine needle biopsy of the liver, which found debris consistent with an abscess and no evidence of malignancy. Attempt to drain liver lesions percutaneously failed. He was treated with ceftriaxone for 4 weeks and transitioned to ciprofloxacin for a total of eight weeks of therapy. On repeat imaging 5 months after initial presentation his liver was noted to be unremarkable. |
| 1 | Hepatic abscesses, secondary empyema, and bacteremia | A 76-year-old woman with a history of atrial fibrillation, type B aortic dissection, hypertension, and hypothyroidism presented with syncope and unwitnessed head trauma. She reported having fevers in the week before presentation. She also reported several episodes of pneumonia, treated at other hospitals over the five months prior to presentation. On presentation, she was found to be in septic shock requiring vasopressors. CT of the abdomen showed multiple hepatic cysts. | Patient was admitted to the ICU and started on piperacillin-tazobactam. Blood cultures grew hvKp, and treatment was switched to ceftriaxone 2g daily and metronidazole. On hospital day 5, the primary liver lesion was drained percutaneously, the purulent drainage grew hvKp. Repeat abdominal ultrasound on hospital day 11 revealed new complex pulmonary effusion which appeared to be due to the hepatic drain crossing into the pleural space. Chest tube was placed, with 1290cc fluid drained, and hepatic drain removed. The patient was discharged on ertapenem for six weeks from chest tube removal and recovered well. |
| 2 | Hepatic abscess, urinary tract infection, and bacteremia | A 64-year-old woman with a history of chronic hepatitis B and remote colon cancer presented with 5 days of abdominal pain associated with eating. She also endorsed fevers, chills, and night sweats. She denied diarrhea, nausea, or vomiting.  CT of her abdomen revealed hepatic pyogenic abscess. | Hepatic abscess was drained percutaneously, after which the patient was admitted and started on vancomycin and piperacillin-tazobactam. On hospital day 2, blood, urine, and liver abscess cultures grew hvKp. Antibiotics were switched to ceftriaxone 2g daily. She was discharged on hospital day 9 with ciprofloxacin 500mg BID; plan was to base length of course on repeat imaging but was lost to follow up. |
| 13 | Cavitary pneumonia in the setting of severe COVID-19 | A 50-year-old man with a history of impaired fasting glucose presented after collapsing. He reported dry cough, congestion, and dyspnea for two weeks prior to presentation. He was found to be hypoxic with peripheral arterial saturations of 80% on room air and tested positive for SARS-CoV-2 with a nasopharyngeal PCR test. He was diagnosed with acute hypoxic respiratory failure due to COVID-19 pneumonia. | Patient was admitted to the ICU on high flow nasal cannula and treated with dexamethasone, remdesivir, sarilumab, and tocilizumab. He initially improved, however on hospital day 6 his respiratory status worsened, requiring intubation, proning, and neuromuscular blockade. Chest imaging at the time of intubation was consistent with COVID-19 pneumonia without evidence of superimposed bacterial or fungal infection. However, over the next 48 hours his ventilatory requirements worsened, and thoracic computed tomography revealed two cavitary lesions in the bilateral lower lobes measuring up to 57x29mm, favored to represent superimposed atypical or fungal pneumonia. Endotracheal cultures grew *hvKp*, blood cultures were negative*.* He was treated with cefepime 2g every 8 hours and vancomycin. Over the following week his shock and ARDS worsened, ultimately with irreversible respiratory acidosis, and he expired. |
| 3 | Pyelonephritis and hepatic abscess | A 52-year-old woman with no significant medical history presented to the ED with fever, cough, fatigue, nausea, and vomiting for a week. She was afebrile and tachycardic, with a leukocytosis and significant pyuria. She received 1g ceftriaxone and was discharged with cephalexin 500mg four times daily for 7 days. Urine culture later grew hvKp. Symptoms did not improve, she return to ED 9 days later where she was febrile with a leukocytosis, elevated lactate and elevated alkaline phosphatase. Urinalysis was without significant pyuria. | Patient was admitted and ertapenem 1g administered. CT of the abdomen showed 6.9 x 5.2 x 5.9 cm heterogeneous, multiloculated fluid collection in the right hepatic lobe with surrounding edema, compatible with an abscess. The abscess was drained percutaneously with 30cc purulent fluid drained and sent for culture, which grew hvKp. Blood cultures were negative. She was discharged with ceftriaxone 2g daily with a plan to complete 4-6 weeks therapy. |
| 4 | Orbital cellulitis with posterior globe abscess | A 76-year-old woman with a history of hepatitis C complicated by cirrhosis, hemorrhagic stroke with residual left hemiparesis, hypertension, and anemia presented to an outside hospital with left eye pain, swelling, and impaired vision with headache that had lasted for four days. CT orbits with contrast revealed preseptal and postseptal left orbital cellulitis with mild left orbit proptosis. She received vancomycin and piperacillin-tazobactam. Blood cultures grew Kp. She was transferred to our hospital for retinal and oculoplastics specialty care. | She was found to be septic, with left orbital cellulitis, orbital abscess and panophthalmitis, and started on ceftriaxone 2g twice daily given optic nerve involvement. B scan of the left eye revealed large multifocal subretinal abscesses. The right eye remained unaffected. She underwent left orbital evisceration and abscess drainage, where purulence was noted intraoperatively with some necrotic tissue. The bacterial cultures from the eye surgical swab grew hvKp. CSF cultures were negative. CT scan revealed multiple lesions in a cirrhotic liver. A liver biopsy revealed hepatocellular carcinoma with no evidence of abscess. Her hepatocellular carcinoma was found to be metastatic and her family opted for palliative therapy. |
| 5 | Respiratory colonization | A 43-year-old man with a history of methamphetamine use and homelessness was brought in by EMS as a code trauma after he was hit by a car. On presentation he was awake and agitated, found to have multiple complex calvarial fractures and multiple subdural hemorrhages. | He was admitted in the ICU and started on cefepime and vancomycin empirically in the setting of cerebrospinal fluid leak. On hospital day 2, he was intubated. Endotracheal cultures grew hvKp, however chest imaging was clear and ventilatory requirements minimal, and antimicrobial therapy was not changed. |
| 6 | Recurrent pneumonia | A 56-year-old man with a history of stage IV lung adenocarcinoma presented with worsening dyspnea and wheezing and underwent outpatient bronchoscopy with tumor debulking and dilation of bronchi, which alleviated his symptoms. One month later, he again developed symptoms and prescribed azithromycin for 3 days which did not alleviate his symptoms. He underwent repeat bronchoscopy with repeat debulking and stent placement which again alleviated symptoms. Bronchoalveolar lavage grew hvKp, which was not treated. | One month later he again developed symptoms and again underwent bronchoscopy which revealed tumor infiltration of the bronchial stent, as well as thick secretions and mucosal inflammation. Chest x-ray revealed clear lungs. Cultures again grew hvKp, which was treated with 10 days of amoxicillin-clavulanate. A fourth scheduled bronchoscopy showed improved secretions but bronchoalveolar lavage again grew hvKp, which was again treated with amoxicillin-clavulanate for 14 days. He remained outpatient, receiving palliative chemotherapy. |
| 7 | Neck abscess | A 48-year-old man with a history of poorly controlled type 2 diabetes mellitus (A1c 12.3%) presented with a draining neck abscess developing over a month. He was initially told at an outside facility that the swelling represented a necrotic malignancy. Four days prior to presentation it opened and started draining. | Patient was admitted, underwent incision and drainage of the neck abscess, and biopsy was obtained. He was started  on  piperacillin-tazobactam and vancomycin. The drainage grew hvKp and treatment was switched to ceftriaxone 2g q24h. The was no evidence of malignant cells in the biopsy tissue. He was discharged on hospital day 10 with 2 weeks of trimethoprim-sulfamethoxazole. |
| 12 | Infection of right eye | A 30-year-old man with a history of poorly controlled type 1 diabetes mellitus and methamphetamine and heroin injection presented with one week of progressive right eye and ear pain, and loss of vision. He was found to have otitis externa complicated by tympanic membrane perforation, otitis media and mastoiditis, a left parotid abscess, and right endophthalmitis with subretinal abscess. | Patient was admitted and started on piperacillin-tazobactam and vancomycin. Cultures from the left parotid abscess and right ear drainage grew hvKp. He received intravitreal injections of vancomycin, ceftazidime, and voriconazole every other day in addition to systemic antibiotics for two weeks. He was discharged on hospital day 15 but did not fully recover his vision. |
| 8 | Pelvic abscess with bacteremia | A 52-year-old woman with a history of poorly controlled type 2 diabetes mellitus and hypertension presented with urinary retention and worsening pelvic pain in the setting of a known pelvic mass concerning for ovarian malignancy seen at an outside hospital three months prior. She also endorsed several recent UTIs. Two days prior to presentation, she again developed difficulty urinating with worsening pelvic and abdominal pain. She ultimately was completely unable to urinate, which prompted her to present. CT showed a multiseptated 12.2cm pelvic mass, favored to represent a left ovarian neoplasm. | Patient was admitted and started on cefoxitin and doxycycline. On hospital day 2, admission blood cultures grew hvKp and antibiotics were switched to ceftriaxone 2g daily. On hospital day 7, she underwent exploratory laparotomy and 500cc purulent fluid was drained from the uterus. Pathology was consistent with abscess without evidence of malignancy. Ceftriaxone was continued for two weeks inpatient and she was discharged with ciprofloxacin and metronidazole for three more weeks. She was seen outpatient and recovered well. |
| 9 | Pneumonia | A 79-year-old woman with a history of hypertension, cerebrovascular accident, and chronic obstructive pulmonary disease presented with dyspnea and wheezing. She was found to be septic and hypoxic with lactic acidosis, rhabdomyolysis, and leukocytosis. Thoracic CT showed multilobar ground glass opacities. | Patient was admitted to the ICU and started on piperacillin-tazobactam. On hospital day two she was intubated. Bronchoalveolar lavage grew hvKp. Over the next two days she developed renal failure and worsening respiratory failure and expired. |
| 14 | Hepatic abscess with bacteremia | A 39-year-old man with no medical history presented with suprapubic pain, nausea and weakness. He was found to be septic. Abdominal ultrasound demonstrated left hepatic abscesses as well as a distended gallbladder without obstructing stones or evidence of acute cholecystitis. | The patient was admitted and started on piperacillin-tazobactam. Blood cultures grew hvKp and antibiotics switched to ceftriaxone 2g daily. The hepatic abscess was drained percutaneously, he completed 4 weeks of ceftriaxone followed by 2 weeks of trimethoprim-sulfamethoxazole and made a full recovery. |
| 10 | Hepatic and perigastric abscesses | An 82-year-old man with no medical history presented with 2 weeks of fevers, right upper quadrant pain, nausea, and anorexia with weight loss. He was found to have a leukocytosis and elevated transaminases, bilirubin, and alkaline phosphatase. Abdominal CT showed rim-enhancing hypodensities throughout the liver and reactive gallbladder wall thickening with fundal adenomyomatosis, favored to represent cholangitis and micro-abscesses. It also revealed a rim-enhancing fluid collection along the greater curvature of the stomach measuring 6.9 cm. | Patient was admitted and started on piperacillin-tazobactam. ERCP with cholangiogram was normal. He underwent EGD/EUS which revealed a 2.5 cm perigastric abscess a 3-4mm duodenal fistula, and severe gallbladder wall thickening. Biopsies were negative for malignancy. Cultures grew hvKp and *Candida krusei*. Antibiotics were switched to ceftriaxone, metronidazole, and caspofungin for one-week inpatient, he was then discharged with a plan for 4 more weeks of ciprofloxacin, metronidazole, and Isavuconazole. The patient stopped outpatient antibiotics after 7 days and made a full recovery. |
