# Supplementary material for: Clinical and genomic characterization of hypervirulent Klebsiella pneumoniae (hvKp) infections via passive surveillance in Southern California, 2020–2022
Source: Front Microbiol. 2022 Oct 14;13:1001169. doi: 10.3389/fmicb.2022.1001169 (PMC9614223; doi:10.3389/fmicb.2022.1001169)
Supplement: Supplementary file 4 [file Table_2.docx]

**Table S2.** Detailed genomic analysis results of hvKp isolates.

|  |  | U590 | U591 | U613 | U664 | U824 | U885 | U509 |
| --- | --- | --- | --- | --- | --- | --- | --- | --- |
| **Case Study** |  | 15 | 11 | 1 | 2 | 13 | 3 | 4 |
| **Reference Genome** |  | F0834906 | F0834906 | AP006725 | AP006725 | LR745045 | AP006725 | AP006725 |
| Coverage |  | 92.5 | 97.8 | 99.3 | 99.9 | 96.6 | 98.9 | 99.5 |
| Pairwise Identity |  | 98.1 | 98.2 | 99.0 | 94.3 | 98.2 | 98.6 | 98.9 |
| Mean depth (X) |  | 107 | 90 | 88 | 28 | 164 | 115 | 134 |
|  |  |  |  |  |  |  |  |  |
| **Reference Plasmid** |  | F0834905 | F0834905/F0834904 | AP006726 | AP006726 | AP006726 | AP006726 |  |
| Coverage |  | 91.0 | 100/100 | 99.3 | 100 | 95.5 | 99.8 |  |
| Pairwise Identity |  | 98.2 | 97.4/98.0 | 98.8 | 93.7 | 98.7 | 98.8 |  |
| Depth |  | 138 | 138/210 | 75 | 26 | 267 | 134 |  |
| **Capsular Type** |  |  |  |  |  |  |  |  |
| KL Type |  | K2 | K2 | K1 | K1 | K2 | K1 | K1 |
| wzi allele |  | 203 | 257 | 1 | 1 | 2 | 1 | 1 |
| **MLST** |  |  |  |  |  |  |  |  |
| ST |  | 380 | 66 | 23 | 23 | 86 | 23 | 23 |
| **Virulence Factor - P** | **Locus** |  |  |  |  |  |  |  |
| Aerobactin | ***iucA*** |  |  |  |  |  |  |  |
| Salmochelin | ***iroB*** |  |  |  |  |  |  |  |
| Putative transporter | ***peg-344*** |  |  |  |  |  |  |  |
| Regulators of the mucoid phenotype | ***rmpA*** |  |  |  |  |  |  |  |
|  | ***rmpA2*** |  |  |  |  |  |  |  |

|  |  | U838 | U847 | U848 | U353 | U834 | U959 | U937 | U907 |
| --- | --- | --- | --- | --- | --- | --- | --- | --- | --- |
| **Case Study** |  | 5 | 6 | 7 | 12 | 8 | 9 | 14 | 10 |
| **Reference Genome** |  | AP006725 | AP006725 | AP006725 | F0834906 | AP006725 | AP006725 | CP065401 | AP006725 |
| Coverage |  | 98.9 | 99.2 | 99.0 | 99.7 | 99.2 | 99.2 | 98.6 | 99.1 |
| Pairwise Identity |  | 98.2 | 98.3 | 98.7 | 98.8 | 98.6 | 99.0 | 99.2 | 98.7 |
| Mean depth (X) |  | 156.1 | 116.9 | 136.8 | 104.7 | 163.8 | 114.6 | 61.3 | 104.4 |
|  |  |  |  |  |  |  |  |  |  |
| **Reference Plasmid** |  | AP006726 | AP006726 | AP006726 | F0834905/ F0834904 | AP006726 | AP006726 | AP006726 | AP006726 |
| Coverage |  | 99.8 | 99.8 | 99.4 | 100/100 | 100 | 99.4 | 99.1 | 100 |
| Pairwise Identity |  | 98.0 | 98.3 | 98.6 | 98.0/98.7 | 98.4 | 98.7 | 82.4 | 98.6 |
| Depth |  | 155.8 | 153.6 | 161.9 | 163.3/285.9 | 183.0 | 127.5 | 44.7 | 107.1 |
| **Capsular Type** |  |  |  |  |  |  |  |  |  |
| KL Type |  | K1 | K1 | K1 | K2 | K1 | K1 | K2 | K1 |
| wzi allele |  | 1 | 1 | 1 | 257 | 1 | 1 | 72 | 1 |
| **MLST** |  |  |  |  |  |  |  |  |  |
| ST |  | 23 | 23 | 23 | 66 | 23 | 23 | 375 | 23 |
| **Virulence Factor - P** | **Locus** |  |  |  |  |  |  |  |  |
| Aerobactin | ***iucA*** |  |  |  |  |  |  |  |  |
| Salmochelin | ***iroB*** |  |  |  |  |  |  |  |  |
| Putative transporter | ***peg-344*** |  |  |  |  |  |  |  |  |
| Regulators of the mucoid phenotype | ***rmpA*** |  |  |  |  |  |  |  |  |
|  | ***rmpA2*** |  |  |  |  |  |  |  |  |
